# Bioactive Compounds in *Sarcocornia* and *Arthrocnemum*, Two Wild Halophilic Genera from the Iberian Peninsula

**DOI:** 10.3390/plants10102218

**Published:** 2021-10-19

**Authors:** Irene Sánchez-Gavilán, Esteban Ramírez Chueca, Vicenta de la Fuente García

**Affiliations:** Departamento de Biología, Facultad de Ciencias, Universidad Autónoma de Madrid, Cantoblanco, 28049 Madrid, Spain; irene.sanchezgavilan@estudiante.uam.es (I.S.-G.); esteban.ramirez@uam.es (E.R.C.)

**Keywords:** halophytes, salt tolerance, bioactive compounds, flavonoids, fatty acids

## Abstract

(1) Background: this study describes bioactive compounds in the following halophytes: *Sarcocornia* (*S. alpini*, *S. pruinosa*, and *S. perennis*) and *Arthrocnemum* (*A. macrostachyum*). The material comes from: coastal marshes in Tinto River, Guadiana River, and some interior provinces from the Iberian Peninsula. (2) Methods: the techniques used were Folin–Ciocalteu, GC-MS, and ESI-MS/MS. (3) Results: Five phenolic acids were found in *Sarcocornia*: trans-cinnamic, salicylic, veratric, coumaric, and caffeic acids. In addition, in *Arthronemum*, ferulic acid was also detected. The obtained flavonoids were cyanidin-3-O-arabinoside, luteolin-7-glucoside, dihydroquercetin, and p-coumaroyl-glucoside. They also presented fatty acids, such as palmitic, linoleic, and oleic acids in *Sarcocornia*, while palmitic, linolenic, and stearic acids were the main fatty acids in *A. macrostachyum*. (4) Conclusions: the high diversity of the compounds identified confirms the relation between nutritional interest and salt tolerance in halophytes.

## 1. Introduction

The genera *Arthrocnemum* Moq. and *Sarcocornia* A.J. Scott (Chenopodiaceae/Amaranthaceae) include succulent chamaephytes that are specialized in the colonization of saline habitats. In European and North African Mediterranean territories, the following taxa occur: *Arthrocnemum* *macrostachyum* (Moric.) K. Koch; *A. meridionale* Ramírez, Rufo, Sánchez-Mata, and Fuente; *Sarcocornia* *hispanica* Fuente, Rufo, and Sánchez-Mata; and *S. alpini* (Lag.) Rivas-Martínez, *S. carinata* Fuente, Rufo, Sánchez-Mata, and *S. fruticosa* (L.) A.J. Scott. In contrast, *S. perennis* (Mill) A.J. Scott, *S. pruinosa* Fuente, Rufo, and Sánchez-Mata are limited to the European Atlantic coasts [1,2,3,4,5,6].

The Chenopodiaceae species are generally characterized by a high content of minerals, polyphenols, and fatty acids, among other compounds of interest. The abundance of inorganic elements (Na^+^, K^+^, Mg^2+^, and Ca^2+^, among others) in the tissues of these plants, together with the wide diversity of bioactive compounds, have been related to their capacity to survive and grow in extreme environments with high salinity and long periods of intense drought [7,8,9,10].

*Arthrocnemum macrostachyum* has recently been used in soil desalination programs [11] due to its capacity to accumulate high concentrations of sodium chloride in its tissues and hence to reduce it in the cultivation medium. El-Naker et al. [12] recorded the presence of a wide range of phytochemical compounds in this genus and identified sixteen that were potentially bioactive, some of which have antioxidant (quercetin, 4-hydroxybenzoic, and caffeic acids), antiviral, antibacterial, and/or anti-tumoral properties (hesperidin, salicylic, chlorogenic, and coumaric acids), including compounds for the treatment of diabetes (rhamnetin).

Several species of *Sarcocornia* have been evaluated as edible plants due to their different nutritional properties, particularly including their antioxidant capacity and lipid composition. Riquelme et al. [13] characterized different phenolic compounds in *Sarcocornia neei* (Lag.) M.A. Alonso and M.B. Crespo, such as kaempferol and quercetin, as well as gallic, ferulic, and coumaric acids, among others. Barreira et al. [8] analyzed the fatty acid profile of *Sarcocornia* spp. collected in the Algarve (Portugal) and reported a predominance of palmitic, linolenic, and linoleic acids. These same authors also detected and quantified greater quantities of these fatty acids in *Arthrocnemum macrostachyum* from the marshes of Praia de Faro in the south of the country. The sustained implementation of the potential of both genera as emerging quality crops began in the 1990s and has continued to the present [14,15,16,17,18,19].

Our selection of the genera *Sarcocornia* and *Arthrocnemum* was guided by the importance and interest of halophytes in today’s agriculture. There are two factors that make halophytes of special interest to be considered: First, their economic potential, considering their productivity in high-salinity and low-water intake environments, and second, their nutritional value in terms of protein, phenolic, and lipid contents, and the great quantity of minerals such as iron, potassium, calcium, and magnesium, as well as other bioactive compounds [8,10]. Samples of the species *S. alpini*, *S. pruinosa*, and *S. perennis* of the genus *Sarcocornia*, which were all from the southwestern Iberian Peninsula (Spain and Portugal), were analyzed. Within the province of Huelva (Spain), the largest number of samples studied originated from a special area of the marshland influenced by the Tinto River. This territory has an abundance of natural heavy metals (especially Cu, Zn, Cr, and Fe) and a slightly acidic pH (6.27–6.35), specifically in the estuarine area that runs from San Juan del Puerto to the river’s mouth, together with the Odiel river in the Atlantic Ocean [20,21]. Additionally, in this area, the three species of the genus *Sarcocornia* occupy and dominate a large part of the vegetation of the marshes in an ecological gradient strongly marked by the greater or lesser proximity to the sea, as well as by the dryness of the soil: *S. perennis* occurs in the first vegetation band, almost constantly submerged by the tides; *S. pruinosa* appears in an upper band, occasionally influenced by the tides; and, finally, *S. alpini* dominates in soils that are further away from the tidal influence and are drier [4]. In turn, *A. macrostachyum* also dominates in the southwestern Iberian region, in the driest salt marshes with the highest saline concentration and in an ecological zone farther from the sea [22]. Additionally, samples of *A. macrostachyum* from the interior of the Iberian Peninsula have also been analyzed; these substrates undergo strong summer desiccation with the outcrop of saline efflorescence, in addition to increasing their Ca cation content [23]. It has been proven that the greater or lesser exposure to saline conditions of halophytes in their natural environment has an influence on the content of the bioactive phytochemicals present in them, especially as a protection mechanism against the oxidizing agents produced in these extreme environmental conditions [24]. There is great interest in studying the role that compounds play in the adaptation of halophytes to these environments. Thus, the main objective of this work was to determine the bioactive compounds (phenolic compounds and fatty acids) in various species of *Sarcocornia* (*S. alpini*, *S. pruinosa*, and *S. perennis*) and *Arthrocnemum* (*A. macrostachyum*) in coastal and inland saltmarshes of the Iberian Peninsula.

## 2. Results

### 2.1. Total Phenolic Compounds (TPC) and Phenolic Acids

**Figure 1:**
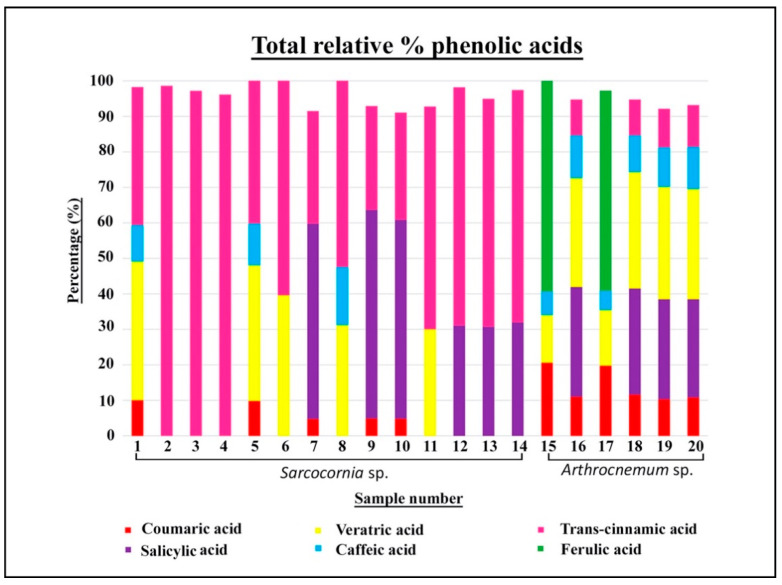
Total relative percentages of the phenolic acids.

**Figure 2:**
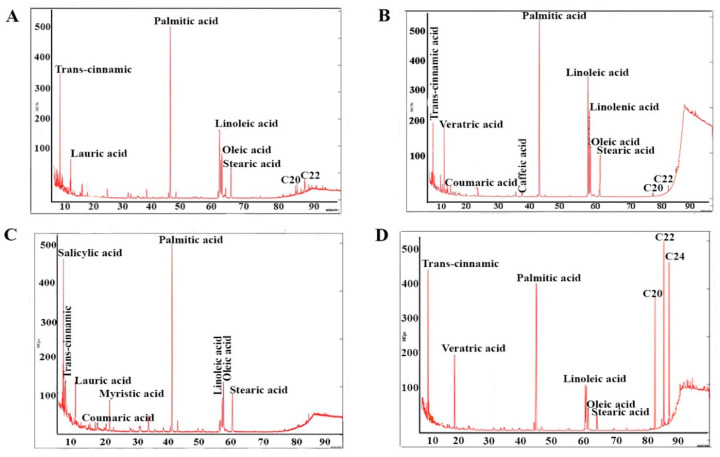
Chromatograms of the samples: phenolic acids and fatty acids in *S. alpini* and *S. pruinosa*. (**A**) *S. alpini* samples 2, 3, and 4. (**B**) *S. alpini* samples 1 and 5. (**C**) *S. pruinosa* samples 7, 9, and 10. (**D**) *S. pruinosa* samples 6 and 8.

**Figure 3:**
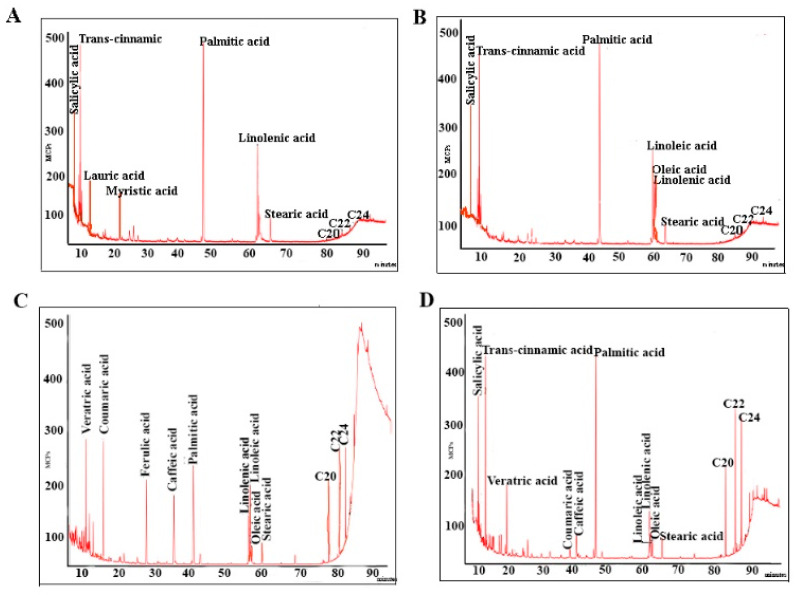
Chromatograms of the samples studied: phenolic acids and fatty acids in *S. perennis* and *A. macrostachyum* (**A**) *S. perennis* sample 12. (**B**) *S. perennis* samples 13 and 14. (**C**) *A. macrostachyum* samples 15 and 17. (**D**) *A. macrostachyum* samples 16 and 18–20.

Our values for the total phenolic compounds in the genus *Sarcocornia* expressed as gallic acid equivalent (G.A.E.) were between 3.892 mg G.A.E./g plant dw (dry weight) and 3.231 mg G.A.E./g plant dw (Table 1). The phenolic acids found in the species of *Sarcocornia* and *Arthrocnemum* were benzoic acids (salicylic and veratric) and hydroxycinnamic acids (trans-cinnamic, caffeic, coumaric, and ferulic). All the material from the genus *Sarcocornia* presented trans-cinnamic acid, which is the most frequent and abundant compound (Table S1, Figure 1, Figure 2 and Figure 3).

Sample 1 of *S. alpini* presented veratric acid and trans-cinnamic acid with 39% of relative content, while coumaric and caffeic acid accounted for around 10%. For *S. alpini* material corresponding to samples 2, 3, and 4, only trans-cinnamic acid was detected between 96% and 98%.

In *S. pruinosa*, sample 6 had a content of 60% of trans-cinnamic acid and 39% of veratric acid. Sample 7 contained 60% salicylic acid, 32% trans-cinnamic acid, and a minority of coumaric acid with 4%. Samples 9 and 10 had salicylic acid between 56% and 58%, trans-cinnamic acid with 30%, and, finally, 4% coumaric acid. Sample 11 presented 62% trans-cinnamic acid and 30% veratric acid.

All the material of *S. perennis* presented trans-cinnamic acid between 64% and 67%, and salicylic acid between 30% and 32%.

The total phenolic compounds in *A. macrostachyum* were between 4.891 mg G.A.E./g plant dw and 4.220 mg G.A.E./g plant dw.

Sample 15 contained 59% ferulic acid, which was notable, 20% coumaric acid, 13% veratric acid, and 5% caffeic acid. Sample 17 had a content of 56% ferulic acid, 19% coumaric acid, 15% veratric acid, and 6% caffeic acid.

Samples 16, 18, 19, and 20 had salicylic acid and veratric acid at 30%, in addition to caffeic acid and trans-cinnamic acid, both at 10%. These samples did not contain ferulic acid.

### 2.2. Flavonoids and Hydroxycinnamic Acids

All the samples of *Sarcocornia* and *Arthrocnemum* studied contained luteolin and this was the only flavonoid present in *S. perennis*. Cyanidin-3-*O*-arabinoside and luteolin-7-glucoside (Table 2 and Table S3) were found in *S. alpini*, while *S. pruinosa* contained dihydroquercetin and p-Coumaroyl tyrosine. *A. macrostachyum* contained dihydroquercetin and p-Coumaroyl-glucoside. The chemical structures of these compounds are shown in Figure S1.

### 2.3. Fatty Acids

**Figure 4:**
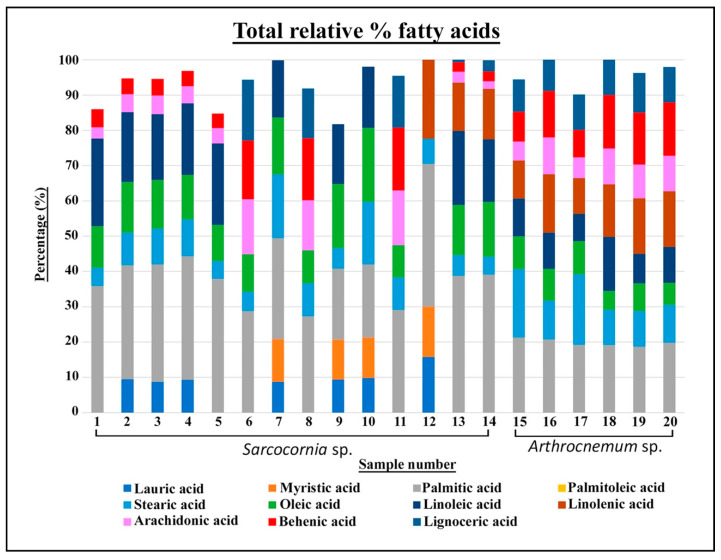
Total relative percentages of the fatty acids.

Our results show that the total proportion of saturated fatty acids in the genus *Sarcocornia* represented a mean of 61.5% relative percentage, with lower proportions of monounsaturated fatty acids at 2.7% (Table S2, Figure 2, Figure 3 and Figure 4). Polyunsaturated fatty acids were at 19.20% relative percentage.

In the genus *Arthrocnemum*, saturated fatty acids represented a mean of 65.2% relative percentage, monounsaturated fatty acids accounted for 7.8% relative percentage, and polyunsaturated fatty acids were 24.1% relative percentage.

Among the saturated fatty acids, the palmitic acid was notable, which was present between 30% and 20% relative percentage in all samples of both *Sarcocornia* and *Arthrocnemum*; this was also the case for stearic acid but with lower percentages between 20% and 10% relative percentage.

Lauric and myristic acids were found only in material from the genus *Sarcocornia*. Lauric acid was notable in *S. pruinosa*, *S. alpini*, and *S. perennis*, with a relative content of between 15% and 8% relative percentage. Myristic acid was detected in *S. alpini* and *S. perennis* with a content of over 10% relative percentage.

Among monounsaturated fatty acids, the study material presented oleic acid with values between 20% and 10%, particularly in *S. alpini* and *S. perennis*.

Linoleic acid was the predominant polyunsaturated fatty acid in the genus *Sarcocornia*, with values between 24% and 18% in *S. alpini*, 17% in *S. pruinosa*, and between 21% and 17% in *S. perennis*. This acid decreased to a relative content of 10% in *A. macrostachyum*.

Finally, linolenic acid was present only in *S. perennis* between 22% and 17%, and in *A. macrostachyum* with a relative content of between 16% and 10%.

Arachidonic and behenic acids were found in all the samples of *A. macrostachyum* and *S. alpini*, with values ranging from 15% to 5%, whereas in *S. pruinosa* and *S. perennis*, the content varied between the different samples. 

Lignoceric acid was identified in the samples of *S. pruinosa* and *S. perennis* from the estuary of the Tinto River (samples 6, 8, 11, 13, and 14), but was absent in *S. alpini*. This fatty acid was found in all the material of *A. macrostachyum* in proportions of between 11% and 9% relative percentage.

## 3. Discussion

### 3.1. Total Phenolic Compounds (TPC)

Our data were within a range of 3.231 to 4.803 mg G.A.E./g plant dw in 20 samples from different populations of *Sarcocornia* and *Arthrocnemum* in the southwest and interior region of the Iberian Peninsula.

Other authors in localities in Portugal have reported values of 20 mg G.A.E./g plant dw for *S. alpini*, of *S. perennis* in populations in Castro Marim, and of 49 mg G.A.E./g plant dw for *A. macrostachyum* collected in Faro, notably both localities in Portugal [8].

This difference can be due to several factors related to the culture conditions of the fresh plant, including the salt stress conditions and environmental changes. Halophytes live in extremely harsh environments with high salinities and UV radiation, and these stressful conditions lead to the production of secondary metabolites such as the phenolic compounds in different concentrations [25]. The mere fact of detecting these total phenolic compounds emphasizes the antioxidant capacity of the halophytes in the study [26,27,28] and demonstrates that *Sarcocornia* and *Arthrocnemum* have a potential food use.

### 3.2. Phenolic Acids

Trans-cinnamic acid was significant in the species of the genus *Sarcocornia,* analyzed particularly in *S. alpini* and *S. perennis*, which were collected in the estuary of the Tinto River and in the mouth of the Guadiana River.

Trans-cinnamic acid was very scarce in *Arthrocnemum macrostachyum*, with a proportion of a little over 10%; the abundance of this acid differs between these genera.

Trans-cinnamic acid reduces adipogenesis and lipogenesis, emphasizing its potential for treating obesity [29].

Salicylic acid was the predominant phenolic acid in *S. pruinosa* with over 55%; however, its content ranged between 30% and 25% in *S. perennis*. Salicylic acid accounted for over 60% of the relative content in the material of *A. macrostachyum* from the Tinto River. This acid is involved in regulating plants’ response to drought through the genetic expression of the genes PR1 and PR2. The induction of these genes increases the accumulation of salicylic acid as a protection mechanism at times of water stress [30].

Veratric acid was significant in the samples that were not affected by the influence of the tides and occupied drier environments in the salt marshes. This was the case of the populations of *S. alpini* and *S. pruinosa* in the southwest of the Iberian Peninsula, and the populations of *A. macrostachyum* in the interior and southwest. Veratric acid has antibacterial, anti-inflammatory, and anti-hypertensive activities [31].

A slightly greater diversity of phenolic acids has been shown more in *A. macrostachyum* than in species of the genus *Sarcocornia*. There is a clear difference in the content of coumaric acid in these genera within the Iberian Peninsula. Five out of fifteen samples of the genus *Sarcocornia* (samples 1, 5, 7, 9, and 10) were identified as having a relative content between 10% and 4%, while this acid was detected in the six samples analyzed of *Arthrocnemum*, with content ranging between 20% and 10%. This acid has been described in *Salicornia patula* Duval–Jouve on the Iberian Peninsula, where it was determined to be infrequent, as it was found in only two samples of the thirteen evaluated [10].

Caffeic acid is the lowest phenolic acid in the *Sarcocornia* material, as indicated by other authors such as Bertin et al. [32] and Costa et al. [33], with data of 0.402 mg/g in *S. ambigua* from Brazil. This acid thickens the plant cell walls and increases resistance to the ionic toxicity of sodium and heavy metal stress [34], suggesting that the presence of caffeic acid in these halophytes may allow them to adapt to highly saline environments.

Ferulic acid was only identified in *A. macrostachyum* collected in the localities of La Rábida and Belchite, with contents of between 59% and 56%. This phenolic acid has been described in *S. ambigua* [33] and *Salicornia europaea* L. [35]. Deng et al. (2015) observed a positive correlation between the ferulic acid content in the cuticle of *Limonium bicolor* (Bunge) Kuntze and the speed of sodium secretion, suggesting that ferulic acid is directly involved in the secretion of salt through saline glands [36]. These glands have not been described in species of *Arthrocnemum* and *Sarcocornia* [3,37]; however, the detection of ferulic acid in two of our populations of *Arthrocnemum* may point to its implication in certain mechanisms of tolerance to salinity.

Additionally, it has been proven that plants exposed to environments with heavy metals produce a high diversity of secondary metabolites, such as phenolic acids [38]. In our study, the diversity of phenolic acids found seems to correspond more closely with the plant species used (*S*. *alpini*, *S. pruinosa*, *S. perennis*, and *A. macrostachyum*) rather than with the influence of a medium with a high content of heavy metals, such as the Tinto River. However, the subject really deserves a detailed study in this regard, especially concerning wild plants that grow under the influence of the Tinto River in the province of Huelva.

### 3.3. Flavonoids and Hydroxycinnamic Acids

All the samples of *Sarcocornia* presented luteolin, which was previously identified in other Salicornioideae such as *S. europaea* [39].

Most flavonoids are present in plants in the form of esters, glucosides, and polymers. The chemical structure of these flavonoids determines their range of intestinal absorption and confers their beneficial uses for halophytes as edible plants. Glycosylation guarantees selective absorption and endows these compounds with prebiotic actions [40]. The species of *Sarcocornia* studied, namely *S. pruinosa* and *S*. *alpini*, also contained a glycosylated flavonoid with greater molecular weight (cyanidin-3-*O*-arabinoside, luteolin-7-glucoside, and dihydroquercetin). p-Coumaroyl glucose was found in *Arthrocnemum*. The presence of these compounds could be explained by the fact that halophytes increase their antioxidant requirements as a defense against extreme environments, forming macromolecular antioxidants [41]. The detection of these compounds also highlights the value of their use as edible plants.

In addition, apigenin-7-glucoside or rutin were identified in the 20 samples of the analyzed genera *Sarcocornia* and *Arthrocnemum* from material from the Iberian salt marshes; these two antioxidant compounds were identified in the genus *Salicornia* and, in the case of rutin, are associated to its tolerance of salinity [10].

### 3.4. Fatty Acids

Fatty acid composition affects the ability to tolerate salt stress [42,43]. Ten different fatty acids were found in the samples from the genus *Sarcocornia* and eight in *Arthrocnemum*. These included saturated, monounsaturated, and polyunsaturated fatty acids that provide halophytes an adaptive advantage, as they prevent the oxidative damage caused by the saline stress that is habitual in these environments [24].

Our results show that the saturated fatty acid present in the highest proportion in all species of *Sarcocornia* and *A. macrostachyum* was palmitic acid, which may account for over 90%. Values of 20% were found in other species of *Sarcocornia*, such as *S. ambigua* [44] and in *Arthrocnemum* from Tunisia, with a content of between 19% and 11% [45]. Stearic acid was another important acid that was present in all the halophyte samples studied, with values ranging from 19% to 5%. Other authors have reported similar results between 18% and 12% in *Sarcocornia* from Alcochete in Portugal [46]. Custodio et al. [47] identified 6% stearic acid in *A. macrostachyum*, which was also collected in Faro, Portugal. These bioactive compounds prevent the development of cardiovascular disorders, reduce insulin resistance, and strengthen the immune system [48].

No palmitoleic acid was found in the material from *Sarcocornia* and *Arthrocnemum* in our study. This monounsaturated fatty acid has been identified by other authors in *S. perennis* and *S. alpini* from Portugal, with values of between 21% and 17% [8]. These authors describe a content of between 6% and 4%, while Custodio et al. [47] reported values of between 11% and 4% in *A*. *macrostachyum* from Portugal.

There was a notable content of polyunsaturated fatty acids, specifically linoleic and linolenic acid. in *S. perennis* and *A. macrostachyum*, with a content of between 22% and 7%, which was much higher than the value described by Barreira et al. [8] in *S. perennis*, namely between 2% and 0.81%. This group of fatty acids have been considered the most important compounds against saline stress and their action has been proposed as an antioxidant [49]. Polyunsaturated fatty acids are bioactive compounds with antifungal activity, in addition to inhibiting carcinogenesis and the progression of atherosclerosis [50].

Long-chain fatty acids, such as arachidonic, behenic, and lignoceric acid, have content values of 17% in the species studied from the genus *Sarcocornia*; these values are higher than those published by Barreira et al. [8], who reported data between 11% and 4% for *S. perennis* and *S. alpini*, for populations from Portugal.

In *A. macrostachyum*, long-chain fatty acids had a relative content of over 15%, notably higher than the values published by Barreira et al. [8] and Custodio et al. [47] for the same species in Portugal.

### 3.5. Nutritional Importance and Future Implications

Halophytic plants of the Salicornioideae subfamily are known as “sea asparagus”, “glasswort”, “samphire”, and “pickleweed” [51,52]. The plants most consumed as gourmet foods are those annual species of the genus *Salicornia,* especially those named under *S. europaea*, which may include other species given the taxonomic complexity of this group [53]. In fact, the difficulty of distinguishing between these types of plants has led many European markets and restaurants to use these halophytes as a mixture of several species, both annual and perennial [18,51].

Perennial halophytes, such as some species of the genera *Sarcocornia* and *Arthrocnemum* (evolutionarily close to *Salicornia*), have also shown to possess interesting nutritional properties for consumption [8,14,19]. *Sarcocornia* and *Arthrocnemum* produce succulent shoots which can be used for food as green leafy vegetables, as fresh ingredients for salads, and for spicing or substituting salt considering their great sodium amounts [48].

In our study, we analyzed the phenolic compounds and fatty acids of perennial plants of the genera *Sarcocornia* (*S. alpini*, *S. perennis* and *S. pruinosa*) and *Arthrocnemum* (*A. macrostachyum*), reaffirming that they are halophytes that also present properties with authentic nutritional potential for its consumption (potential foods with antioxidant properties, contribution of essential fatty acids for the human diet, etc.). The selective introduction of these underused species in markets and in traditional and healthy cuisine represents a future challenge to be implemented.

## 4. Materials and Methods

### 4.1. Materials

The material was collected in the southwest of the Iberian Peninsula in several localities in the Tinto River basin, such as La Rábida, San Juan del Puerto, and the river estuary. Samples were also collected in localities in the mouth of the Guadiana River (Ayamonte and Castro Marim) and in other points of southeast Portugal (Tavira and Santa Luzia). Salt marshes in Madrid and Zaragoza were selected from the interior of the Iberian Peninsula.

Table 3 shows the data for each of the species studied and fresh plants were collected as follows: Upon reception, a portion of fresh plants was stored in airtight plastic bags (anaerogen ^TM^ 3.5 L, Thermo Scientific, Waltham, MA, USA) for one day until its analyses were performed. Then all material were dehydrated in a recirculated air stove (MEMMERT) to 100 °C for six hours for the subsequent analysis of the bioactive compounds: *Sarcocornia alpini* (1–5), *Sarcocornia pruinosa* (6–11), *Sarcocornia perennis* (12–14), and *Arthrocnemum macrostachyum* (15–20).

### 4.2. Methods

#### 4.2.1. Determination of Humidity

Humidity was determined by drying in an oven (984.25-AOAC, 2005): 5 g of dried samples were weighed in previously dried and tared capsules, and it was placed in a dryer. The samples were placed in a recirculated air stove (MEMMERT) to 100 °C for six hours until the elimination of the water present in the sample (constant weighing). 

#### 4.2.2. Preparation of Methanol Extract

Five hundred milligrams of dried plant sample were extracted with a solution of 40 mL of methanol at 25 °C. It was kept in magnetic stirring for 60 min. The extracts were filtered using a Whatman No. 4 filter. The solid residue was recovered and extracted with 40 mlf of methanol. The extracts were filtered again, combined, and evaporated (35 °C under vacuum of the methanolic extracts). Redissolve with methanol to obtain a 30 mg/mL of extract solution, from which different dilutions were made (from 0.03125 mg/mL to 16 mg/mL), was conducted. The extractions were performed in triplicates and were stored at 4 °C until the execution of the analyses.

#### 4.2.3. Total Phenolic Compounds (TPC)

The total phenolic content was determined by the Folin–Ciocalteu method [54] using gallic acid as the recommended standard [55]. An 0.5 mL of aliquot of methanolic extract was taken from the extracts obtained previously and 2.5 mL of the Folin–Ciocalteu reagent was added and left to react for 3 min. Then, 2 mL of Na_2_CO_3_ solution was added and mixed in a Heidolph shaker (Berlin, Germany). The solution was incubated at a temperature of 40 °C and stored in the dark for 1 h. The absorbance was measured at 765 nm with a spectrophotometer and the results were expressed as gallic acid equivalents (G.A.E.).

#### 4.2.4. Gas Chromatography Coupled with Mass Spectrometry (GC-MS) for Phenolic and Fatty Acid Analysis

Chromatographic separation was performed as follows: Methanol extracts were brought to dryness in a Rotavapor Fischer rotary evaporator (USA) and later in the Telstar lyophilizer (Barcelona, Spain). The amount of the total sample obtained was weighed. The samples were then subjected to derivatization with a 0.2 N methanolic solution of m-trifluoromethylphenyl trimethylammonium hydroxide Meth Prep II (Fisher, Loughborough, UK). This one-step reagent simplifies the transesterification of triglycerides to methyl esters. In total, 5 µL was injected into GC/MS Agilent 6120 (Santa Clara, CA, USA). All standards were from Sigma Aldrich (Sant Louis, MI, USA) at ≥95.0% (HPLC).

The chromatography-mass spectrometry was carried out with the Interdepartmental Research Service at the Universidad Autónoma de Madrid (UAM).

#### 4.2.5. High-Performance Liquid Chromatography-Electrospray Ionization Mass Spectrometry (HPLC-MS/ESI) for Flavonoid and Hydroxycinnamic Acid Analysis

Flavonoids were determined using a HPLC-MS/ESI Agilent 1100 (Santa Clara, CA, USA) in a C20 column ACE 3 C18 PFP, 150 mm × 4.6 mm, which was maintained at 35 °C. The solvent system used was a gradient of water (solvent A) and formic acid 0.1% (solvent A), and the acetonitrile and formic acid 0.1% (solvent B) as follows. For solvent A: 0 min, 96% of solvent A; 10 min, 90% of solvent A; 20 min, 80% of solvent A; 35 min, 60% of solvent A; 40 min, 40% of solvent A; 45 min, 40% of solvent A; 55 min, 96% of solvent A; and 60 min, 96% of solvent A. For solvent B: 0 min, 4% of solvent B; 10 min, 10% of solvent B; 20 min, 20% of solvent B; 35 min, 40% of solvent B; 40 min, 60% of solvent B; 45 min, 60% of solvent B; 55 min, 4% of solvent B; and 60 min, 4% of solvent B. The flow rate was 0.5 mL/min and runs were monitored with an ESI detector set at 280 nm (phenolic acids) and 360 nm (flavonols) for a total chromatogram time of 50 min. The fragmenter worked with 100 V. The drying gas flow was 10 L/min. The nebulizer pressure was 40 psig. The drying gas temperature was 350 °C. The vaporizer temperature was 250 °C. The capillary voltage was 4000 V. The charging voltage was 2000 V. An injection volume of 10 µL was taken from 1.2 mg/2 mL. This technique was used to identity the flavonoids in the extract according to their protonation [M+H] and to calculate the relative retention time of each peak in the chromatograms obtained by HPLC, with a mass range from 700 to 600 umas.

## 5. Conclusions

The bioactive compounds (phenolic compounds and fatty acids) present in *S. alpini*, *S. pruinosa*, *S. perennis*, and *A. macrostachyum* from different territories of Spain and Portugal were described. Samples of the genus *Sarcocornia* highlighted the presence of veratric acid material from dryer environments. A slightly greater diversity of phenolic acids was shown in *A. macrostachyum* than in species of the genus *Sarcocornia*. Ferulic acid was also detected in two of the samples from this genus but was not present in the genus *Sarcocornia*. The composition of the flavonoids detected in these species showed glycosylated structures that conferred prebiotic properties of these halophytes. The material from *S. alpini*, *S. pruinosa*, and *A. macrostachyum* contained macromolecular antioxidants, namely cyanidin-3-*O*-arabinoside, luteolin-7-glucoside, dihydroquercetin, and p-Coumaroyl glucoside, thus increasing their antioxidant requirements as a defense against extreme environments. The lipid profile revealed palmitic, linoleic, and oleic acids as the main fatty acids in the genus *Sarcocornia*, while the palmitic, linolenic, and stearic acid content was particularly notable in the genus *Arthrocnemum*. The presence of these compounds in different halophytes confirms their value for survival in conditions of extreme salinity and drought, and also adds to their value for consumption.

## Figures and Tables

**Table 1:** Data on the sample weight and total phenolic compounds (TPC ± SD (mg G.A.E./g plant dw)) for dry material and humidity. Note: gallic acid equivalent (G.A.E.) and standard deviation (SD). *n* = 3.

ID	Sample	TPC ± SD	Humidity
1	*S. alpini*	3.611 ± 0.107	10.23%
2	*S. alpini*	3.569 ± 0.233	10.59%
3	*S. alpini*	3.778 ± 0.231	11.80%
4	*S. alpini*	3.480 ± 0.164	10.40%
5	*S. alpini*	3.430 ± 0.093	11.30%
6	*S. pruinosa*	3.892 ± 0.203	10.18%
7	*S. pruinosa*	3.879 ± 0.207	10.53%
8	*S. pruinosa*	3.404 ± 0.198	14.78%
9	*S. pruinosa*	3.453 ± 0.064	14.44%
10	*S. pruinosa*	3.299 ± 0.156	13.17%
11	*S. pruinosa*	3.231 ± 0.089	13.65%
12	*S. perennis*	3.407 ± 0.004	10.29%
13	*S. perennis*	3.420 ± 0.139	13.16%
14	*S. perennis*	3.460 ± 0.014	12.35%
15	*A. macrostachyum*	4.680 ± 0.036	10.35%
16	*A. macrostachyum*	4.891 ± 0.060	11.80%
17	*A. macrostachyum*	4.803 ± 0.096	11.36%
18	*A. macrostachyum*	4.220 ± 0.014	10.55%
19	*A. macrostachyum*	4.850 ± 0.012	13.71%
20	*A. macrostachyum*	4.770 ± 0.005	13.64%

**Table 2:** Tentative identification of flavonoids in *Sarcocornia* and *Arthrocnemum*.

Species	Flavonoid Compound	Experimental Mass M-H m/z	MS/MS (m/z)
*S. alpini*, *S. pruinosa*, *S. perennis*, *A. macrostachyum*	Luteolin	287	285/290
*S. alpini*	Cyanidin-3-*O*-arabinoside	419	415/422
	Luteolin-7-glucoside	448	446/450
*S. pruinosa and A. macrostachyum*	Dihydroquercetin	304	303/310
*S. pruinosa*	p-Coumaroyl tyrosine	327	322/330
*A. macrostachyum*	p-Coumaroyl-glucoside	326	322/330

**Table 3:** Data on the material: geographic locations, collection date, and MGRS (Military Grid Reference System) of the *S. alpini*, *S. pruinosa*, *S. perennis*, and *A. macrostachyum* samples from the Iberian Peninsula (Spain and Portugal).

ID	Sample	Geographic Location	Collection Date	MGRS Coordinates
1	*S. alpini*	Spain, Huelva, and San Juan del Puerto	15 December 2017	29SPB9230
2	*S. alpini*	Spain, Huelva, and La Rábida	17 July 2018	29SPB8320
3	*S. alpini*	Spain, Huelva, and Ayamonte	18 July 2018	29SPB4122
4	*S. alpini*	Portugal and Esteiro de Carrasqueira	18 July 2018	29SPB3918
5	*S. alpini*	Spain, Huelva, San Juan del Puerto, and saltmarshes of the “Embarcadero de Buitrón”	7 August 2019	29SPB9031
6	*S. pruinosa*	Spain, Huelva, and Tinto river estuary	14 December 2017	29SPB8220
7	*S. pruinosa*	Portugal, Tavira saltmarshes, and Santa Luzia	18 July 2018	29SPB1906
8	*S. pruinosa*	Spain, Huelva, and Punta del Moral	18 July 2018	29SPB4717
9	*S. pruinosa*	Spain, Huelva, and Odiel saltmarshes	7 August 2019	29SPB7926
10	*S. pruinosa*	Spain, Huelva, and La Rábida	17 July 2018	29SPB8320
11	*S. pruinosa*	Spain, Huelva, Odiel River, and “Cabeza Alta”	14 December 2017	29SPB8024
12	*S. perennis*	Portugal, Tavira saltmarshes, and Santa Luzia	18 July 2018	29SPB1806
13	*S. perennis*	Spain, Huelva and Tinto river estuary	14 December 2017	29SPB8220
14	*S. perennis*	Spain, Huelva and Punta del Moral,	18 July 2018	29SPB4717
15	*A. macrostachyum*	Spain, Zaragoza, and Belchite	19 January 2017	30TXL8875
16	*A. macrostachyum*	Spain, Madrid, and Colmenar de Oreja	11 July 2018	31TVK5143
17	*A. macrostachyum*	Spain, Huelva, and La Rábida	15 December 2017	29SPB8320
18	*A. macrostachyum*	Portugal, Marismas de Tavira, and Santa Luzia	18 July 2018	29SPB1906
19	*A. macrostachyum*	Spain, Huelva, San Juan del Puerto, and saltmarshes of the “Embarcadero de Buitrón”	7 August 2019	29SPB9131
20	*A. macrostachyum*	Spain, Huelva, and Ayamonte	18 July 2018	29SPB4218

## Data Availability

The data are contained within the article or Supplementary Material.

## Supplementary Materials

The following are available online at https://www.mdpi.com/article/10.3390/plants10102218/s1, Figure S1: Tentative flavonoid compounds identified in *Sarcocornia* and *Arthrocnemum* extracts. All structures are based on Phenol Explorer; Table S1: Relative content of the phenolic compounds (%) in *Sarcocornia* and *Arthrocnemum* samples by GC-MS. Legend: nd: not detected. Numbers 1–20 refer to the materials shown in Table 3; and Table S2: Relative content of the fatty acids (%) in *Sarcocornia* and *Arthrocnemum* by GC-MS. Legend: lauric acid (C12:0); myristic acid (C14:0); palmitic acid (C16:0); stearic acid (C18:0); oleic acid (C18:1); linoleic acid (C18:2); linolenic acid (C18:3); arachidonic acid (C20:0); behenic acid (C22:0); lignoceric acid (C24:0); SFAs (saturated fatty acids); MUFAs (monounsaturated fatty acids); and PUFAs (polyunsaturated fatty acids). nd: not detected. Numbers 1–20 refer to the materials shown in Table 3.
