# Supplementary material for: Bioactive Compounds in Sarcocornia and Arthrocnemum, Two Wild Halophilic Genera from the Iberian Peninsula
Source: Plants (Basel). 2021 Oct 19;10(10):2218. doi: 10.3390/plants10102218 (PMC8538410; doi:10.3390/plants10102218)
Supplement: Supplementary file 1 [file plants-10-02218-s001.zip › plants-1411813-supplementary.pdf]

Table S1. Relative content of the phenolic compounds (%) in *Sarcocornia* and *Arthrocnemum* samples by GC-MS. Legend: nd: not detected. Numbers 1–20 refer to the materials shown in Table 3.

ID	Coumaric Acid	Salicylic Acid	Veratric Acid	Caffeic Acid	Trans-cinnamic Acid	Ferulic Acid
**1**	10.05 (0.01)	nd	39.11 (0.02)	10.22 (0.01)	38.90 (0.03)	nd
**2**	nd	nd	nd	nd	98.65 (0.02)	nd
**3**	nd	nd	nd	nd	97.24 (0.04)	nd
**4**	nd	nd	nd	nd	96.17 (0.02)	nd
**5**	9.88 (0.19)	nd	38.17 (0.07)	11.78 (0.02)	41.65 (0.03)	nd
**6**	nd	nd	39.65 (0.01)	nd	61.12 (0.01)	nd
**7**	4.81 (0.01)	57.22 (0.05)	nd	nd	31.66 (0.07)	nd
**8**	nd	nd	31.23 (0.03)	16.34 (0.02)	44.68 (0.01)	nd
**9**	4.92 (0.03)	58.77 (0.05)	nd	nd	29.21 (0.02)	nd
**10**	4.87 (0.08)	56.04 (0.05)	nd	nd	30.17 (0.02)	nd
**11**	nd	nd	30.06 (0.02)	nd	62.71 (0.04)	nd
**12**	nd	31.09 (0.05)	nd	nd	67.12 (0.01)	nd
**13**	nd	30.77 (0.01)	nd	nd	64.19 (0.03)	nd
**14**	nd	32.05 (0.02)	nd	nd	65.35 (0.03)	nd
**15**	20.60 (0.03)	nd	13.47 (0.01)	6.58 (0.01)	nd	59.89 (0.07)
**16**	11.07 (0.03)	30.85 (0.01)	30.75 (0.04)	12.02 (0.01)	10.09 (0.02)	nd
**17**	19.80 (0.03)	nd	15.67 (0.01)	5.44 (0.01)	nd	56.40 (0.04)
**18**	11.63 (0.03)	29.88 (0.07)	32.81(0.04)	10.34 (0.01)	10.12 (0.03)	nd
**19**	10.34 (0.03)	28.19 (0.04)	31.64 (0.04)	11.10 (0.01)	10.87 (0.05)	nd
**20**	10.81 (0.03)	27.66 (0.01)	31.08 (0.04)	11.90 (0.01)	11.77(0.03)	nd

Table S2. Relative content of the fatty acids (%) in *Sarcocornia* and *Arthrocnemum* by GC-MS. Legend: lauric acid (C12:0); myristic acid (C14:0); palmitic acid (C16:0); stearic acid (C18:0); oleic acid (C18:1); linoleic acid (C18:2); linolenic acid (C18:3); arachidonic acid (C20:0); behenic acid (C22:0); lignoceric acid (C24:0); SFAs (saturated fatty acids); MUFAs (monounsaturated fatty acids); and PUFAs (polyunsaturated fatty acids). nd: not detected. Numbers 1–20 refer to the materials shown in Table 3.

ID	C12:0	C14:0	C16:0	C18:0	C18:1	C18:2	C18:3	C20:0	C22:0	C24:0	Total SFAs	Total MUFAs	Total PUFAs
**1**	nd	nd	35.90	5.11	11.83	24.88	nd	3.15	5.09	nd	49.25	11.83	24.88
**2**	9.45	nd	32.29	9.28	14.39	19.75	nd	5.10	4.44	nd	60.56	14.39	19.75
**3**	8.69	nd	33.24	10.14	13.90	18.60	nd	5.30	4.69	nd	62.06	13.90	18.60
**4**	9.33	nd	35.01	10.43	12.55	20.33	nd	4.90	4.28	nd	63.95	12.55	20.33
**5**	nd	nd	37.85	5.08	10.28	23.12	nd	4.32	4.11	nd	51.36	10.28	23.12
**6**	nd	nd	28.77	5.44	10.67	nd	nd	15.55	16.72	17.24	83.72	10.67	nd
**7**	8.73	12.09	28.64	18	16.21	16.18	nd	nd	nd	nd	67.48	16.21	16.18
**8**	nd	nd	27.33	9.29	9.43	nd	nd	14.12	17.63	14.11	82.55	9.43	nd
**9**	9.39	11.29	20.11	5.81	18.19	16.94	nd	nd	nd	nd	66.03	18.19	nd
**10**	9.82	11.44	20.71	17.76	20.96	17.33	nd	nd	nd	nd	59.73	20.96	17.33
**11**	nd	nd	29.12	9.16	9.22	nd	nd	15.44	17.87	14.62	82.21	9.22	nd
**12**	15.75	14.55	40.06	7.11	nd	nd	22.40	nd	nd	nd	77,97	nd	22.40
**13**	nd	nd	38.71	5.91	14.20	21.02	13.70	3.09	2.75	2.59	53.05	14.20	34.72
**14**	nd	nd	39.12	5.04	15.52	17.80	14.30	2.14	2.80	3.1	52.20	15.52	32.10

**15**	nd	nd	21.30	19.35	9.13	10.75	10.80	5.35	8.40	9.20	63.60	9.13	26.55
**16**	nd	nd	20.78	10.90	9.04	10.30	16.55	10.40	13.25	9.60	61.93	9.04	26.85
**17**	nd	nd	19.25	19.90	9.44	7.77	10.10	5.90	7.75	10.09	62.74	9.44	17.87
**18**	nd	nd	19.11	10.58	5.40	15.33	14.91	10.12	15.07	10.26	65.11	5.40	29.03
**19**	nd	nd	18.70	10.04	7.88	8.32	15.78	9.60	14.70	11.24	73.16	7.88	18.38
**20**	nd	nd	19.80	10.73	6.20	10.22	15.76	10.08	15.22	9.92	64.80	6.20	25.98

Table S3. Main tentative flavonoids and hydroxycinnamic acids in *Sarcocornia* and *Arthrocnemum* according to their retention time. Numbers 1–20 refer to the materials shown in Table 3. Legend: + = presence; in brackets, we show the retention times. . = absence.

ID	p-coumaroyl glucose	Cyanidin-3-*O*-arabinoside	Luteolin-7-*O*-glucoside	Dihydroquercetin	p-coumaroyl tyrosine	Luteolin
**1**	.	+(21)	+(24.7)	.	.	+(37.2)
**2**	.	.	.	.	.	+(37.2)
**3**	.	.	.	.	.	+(37.2)
**4**	.	.	.	.	.	+(37.2)
**5**	.	+(21)	+(24.7)	.	.	+(37.2)
**6**	+(20.4)	.	+(24.7)	+(30)	+(32)	+(37.2)
**7**	+(20.4)	.	+(24.7)	+(30)	+(32)	+(37.2)
**8**	+(20.4)	.	+(24.7)	+(30)	+(32)	+(37.2)
**9**	+(20.4)	.	+(24.7)	+(30)	+(32)	+(37.2)
**10**	+(20.4)	.	+(24.7)	+(30)	+(32)	+(37.2)
**11**	+(20.4)	.	+(24.7)	+(30)	+(32)	+(37.2)
**12**	.	.	.	.	.	+(37.2)
**13**	.	.	.	.	.	+(37.2)
**14**	.	.	.	.	.	+(37.2)
**15**	.	.	.	+(30)	.	+(37.2)
**16**	.	.	.	+(30)	.	+(37.2)
**17**	.	.	.	+(30)	.	+(37.2)
**18**	.	.	.	+(30)	.	+(37.2)
**19**	.	.	.	+(30)	.	+(37.2)
**20**	.	.	.	+(30)	.	+(37.2)

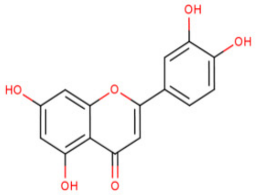

Luteolin

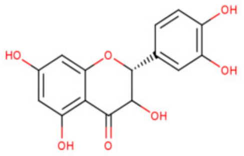

Dihidroquercetin

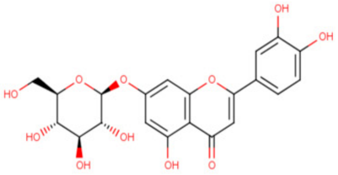

Luteolin-7-O-glucoside

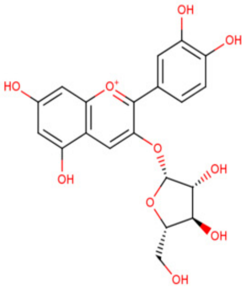

Cianidin-3-O-arabinoside

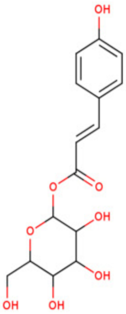

P-coumaroil glucose

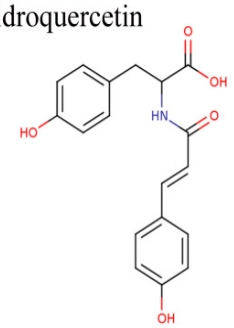

P-coumaroil tirosine

Figure S1. Tentative flavonoid compounds identified in *Sarcocornia* and *Arthrocnemum* extracts. All structures are based on Phenol Explorer.
